# Critical Role of a Survivin/TGF-β/mTORC1 Axis in IGF-I-Mediated Growth of Prostate Epithelial Cells

**DOI:** 10.1371/journal.pone.0061896

**Published:** 2013-05-01

**Authors:** Kyung Song, Eswar Shankar, Jiayi Yang, Kara L. Bane, Reema Wahdan-Alaswad, David Danielpour

**Affiliations:** 1 Case Comprehensive Cancer Center Research Laboratories, The Division of General Medical Sciences-Oncology, Case Western Reserve University, Cleveland, Ohio, United States of America; 2 Department of Pharmacology, Case Western Reserve University, Cleveland, Ohio, United States of America; 3 Department of Biochemistry, Case Western Reserve University, Cleveland, Ohio, United States of America; Florida International University, United States of America

## Abstract

Survivin is a unique member of the inhibitor of apoptosis (IAP) proteins that is overexpressed in numerous cancers through poorly defined mechanisms. One such mechanism may be through constitutive activation of the insulin-like growth factor-I (IGF-I) signaling pathway, implicated in the development and progression of prostate cancer. Using the pre-neoplastic NRP-152 rat prostate cell line as a model, we showed that IGF-I induces Survivin expression, and that silencing Survivin by lentiviral-mediated small hairpin RNA (shRNA) represses IGF-I-stimulated cell growth, implicating Survivin as a mediator of this growth response. Moreover, our data support that the induction of Survivin by IGF-I occurs through a transcriptional mechanism that is mediated in part by the PI3K/Akt/mTORC1 pathway. Use of various Survivin promoter-luciferase constructs revealed that the CDE and CHR response elements in the proximal region of the Survivin promoter are involved in this IGF-I response. Transforming growth factor (TGF-β) signaling antagonists similarly activated the Surivin promoter and rendered cells refractory to further promoter activation by IGF-I. IGF-I suppressed levels of phospho-Smads 2 and 3 with kinetics similar to that of Survivin induction. Suppression of TGF-β signaling, either by TGF-β receptor kinase inhibitors or by silencing Smads 2 and 3, induced Survivin expression and promoted cell growth similar to that induced by IGF-I. TGF-β receptor antagonists also rescued cells from down-regulation of Survivin expression and growth suppression by pharmacological inhibitors of PI3K, Akt, MEK and mTOR. Sh-RNA gene silencing studies suggest that mTORC1 induces while mTORC2 represses the expression of Survivin by IGF-I. Taken together, these results suggest that IGF-I signaling through a PI3K/Akt/mTORC1 mechanism elevates expression of Survivin and promotes growth of prostate epithelial cells by suppressing Smad-dependent autocrine TGF-β signaling.

## Introduction

Survivin (also called BIRC5) is the smallest member of the *i*nhibitor of *ap*optosis (IAP) family of proteins, containing one or more conserved zinc-coordinated Cys/His *b*aculoviral *I*AP *r*epeat (BIR) motifs [1], [2]. While Survivin is well established to block apoptosis elicited by a variety of agents, the mechanism(s) by which it blocks apoptosis is not fully understood [3]. XIAP (an IAP with three BIR motifs) is well established to inhibit apoptosis through binding to caspases, although the overall evidence supporting that Survivin directly inhibits the activity of caspases is not compelling. Rather, studies support that a select pool of Survivin, released from mitochondria upon a death stimulus, inhibits apoptosis by binding to and stabilizing cytosolic XIAP [4] and/or associating to and neutralizing the pro-apoptotic protein Smac/DIABLO [5].

Survivin is a unique mammalian IAP with respect to its function as a mitotic regulator [6]. A significant pool of Survivin resides in the nucleus, where it has been reported to regulate chromosome alignment, chromatin-associated spindle assembly and cytokinesis by physically associating to Auroa B, Borealin and the inner centromere protein (INCENP) [7]. Moreover, Survivin stabilizes the mitotic spindle by binding to polymerized microtubules [8]. Consistent with its vital role in mitosis, expression of Survivin in normal cells is restricted to the G2/M phase of the cell cycle [6], [9]. Such discrete cell-cycle dependent expression is disrupted in tumors, leading to robust elevation of Survivin levels through mechanisms that remain to be resolved. Not surprisingly, Survivin is a putative prognostic marker for a variety of cancers including that of the prostate, breast, lung and colon [3], [10], [11], [12], [13], [14]. Within the nucleus, Survivin has recently been reported to also function as a transcription factor or co-factor, binding to and inhibiting the p21^WAF1/CIP1^ promoter through a p53-dependent mechanism [15]. Histone deaceylase 6 (HDAC6), which can deacetylate Survivin [16], promotes Survivin's nuclear export and subsequently represses its ability to control transcription and mitosis.

The molecular basis for overexpression of Survivin in cancer remains poorly explored. As a regulator of Survivin [17], insulin-like growth factor-I (IGF-I) is a well known survival factor believed to play an important role in the etiology of a variety of cancers [18], [19], [20]. Elevated plasma levels of IGF-I has been shown to predict prostate cancer (PCa) incidence and stage [21]. Importantly, transgenic mice overepressing IGF-I develop PCa [22], and IGF-I receptor neutralizing antibodies repress growth of PCa xenografts [23].

An important negative regulator of Survivin is transforming growth factor-beta (TGF-β [13], [24] a 25 kDa dimeric multifunctional protein that functions through autocrine, paracrine and endocrine modes and regulates a diverse array of cellular and physiological processes such as differentiation, growth suppression, apoptosis, migration, cell survival and epithelial mesenchymal transition [25], [26]. TGF-β signals through a physical association with the ectodomains of two transmembrane serine/threonine kinase receptors known as *T*GF-β *r*eceptor type *II* (TβRII) and TβRI, which upon TGF-β ligand binding form a receptor tetrameric complex. TβRI (also known as Alk5), which is activated through phosphorylation by TβRII kinase, recruits and phosphorylates the two C-terminal serines of Smads 2 and 3. Such phosphorylation exposes their nuclear import sequence, promoting their nuclear localization where they engage in transcriptional control of numerous targets [25], [27], [28].

TGF-β is well recognized to function as a tumor suppressor of the prostate [29], [30], [31], [32], [33], [34], related to its ability to arrest cell growth and/or induce apoptosis of normal or preneoplastic prostate epithelial cells [35]. Our laboratory previously reported that an intact TGF-β signaling pathway transcriptionally downregulates Survivin expression through a mechanism that is dependent on Smads 2 and 3, and two cell cycle repressor elements (within the Survivin proximal promoter), namely a *c*ell cycle-*d*ependent *e*lement (CDE) and a *c*ell cycle genes *h*omology *r*egion (CHR) [36]. TGF-β causes hypophosphorylation of Rb mainly through a Smad3-dependent mechanism, leading to the recruitment of the Rb/E2F4 repressive complex to the CDE/CHR elements of the Survivin promoter. Functional inactivation of Rb family proteins by oncoproteins selectively blocks down-regulation of the Survivin promoter by TGF-β. Moreover, Survivin silencing and overexpression experiments implicate a critical function of this TGF-β response, which is disrupted during tumor progression. Here we provide new evidence that IGF-I functioning predominantly through the phosphatidylinositol 3-kinase (PI3K)/Akt/mammalian target of rapamycin complex 1 (mTORC1) pathway promotes growth of preneoplastic prostate epithelial cells by reversing autocrine TGF-β suppression of Survivin transcription.

## Materials and Methods

### Materials

Sources were: Recombinant human TGF-β1 and anti-Survivin (#AF886) (R&D Systems); anti-P-Smad3 (Ser433/435, #9514), anti-P-Smad2 (Ser465/467, #3101), and P-Smad1/5/8 (Ser463/465/Ser426/428, #9511), anti-mTOR (#2972), anti-Raptor (#2114), anti-Rictor (#4978), anti-P-Rb (Ser807/811, #9308), Akt1 (#2967), Akt (Ser473, #927), anti-P-S6 (Ser235/236, #2211) antibodies (Cell Signaling); anti-Survivin (sc-10811) and anti-Smad3 (sc-8332) antibodies (Santa Cruz); anti-β-actin antibody (Sigma); anti-Smad2 (#S66220) antibody (Transduction laboratories); anti-XIAP (#610762, BD Biosciences); anti-P-Smad3 (Ser423/425) was generous gift obtained from Dr. Dr. Ed Leof; U0126 and rapamycin (LC laboratories), perifosine, Ku-0063794 (Selleck Chem); SB431542 (Tocris Bioscience), SB202190, SP600125, LY294002, HTS-466284 and ALK5 inhibitor-II (EMD Millipore), MK2206 (ChemieTek), DMEM/F12 (Invitrogen), characterized fetal bovine serum (FBS) (HyClone). The rat Survivin promoter-luciferase reporter, sh-Survivin, sh-mTOR, sh-Raptor, and sh-Rictor constructs were developed previously [24], [37]. LNCaP, VCaP, DU145, RWPE-1 and HEK-293T cells were obtained from American Type Culture Collection. HEK-293 cells were obtained from Microbix Biosystems, Inc. (Ontario, Canada).

### Cell culture

NRP-152 prostatic epithelial cell line [38], [39], NRP-152-sh-Smad2, -sh-Smad3, -sh-Smad2+, and -sh-LacZ silencing cell lines [24], [31] were maintained in GM2.1 culture medium as described previously [40]. NRP152-tTR-sh-LacZ and -sh-Survivin, doxycycline-inducible silencing cell lines were cultured in GM2.1 medium. All experiments in NRP-152 and Smad-silencing cell lines were performed in GM3 medium, and experiments involving doxycycline-inducible Survivin silencing cell lines were performed in GM3 medium supplemented with 0.1 µg/ml of doxycycline. LNCaP, VCaP and DU145 were maintained in a 1∶1 mixture of Dulbecco's Modified Eagle's Medium and F12 (DMEM/F12) supplemented with L-glutamine (2 mM), and 5% FBS, and RWPE-1 cells were cultured in keratinocyte growth medium (Invitrogen).

### Development of doxycycline-inducible cell lines, NRP-152-tTR-sh-LacZ and NRP152-tTR-sh-Survivin

Doxycycline-inducible silencing cell lines were developed as previously described [24]. In brief, HEK293T cells were plated at a density of 4×10^6^ cells/10 ml/100 mm dish with 5% FBS+DMEM/F12 medium and transfected with MD2G, PCMV-dr2.74, and PLV-tTR-KREB-Red, using Lipofectamine Plus. Lentiviral supernatants, collected between 24 to 48 h after transfection, were passed through a 0.22 µpore filter, and used to transduce NRP-152 cells. The resulting NRP-152-tTR cells were then infected with lentiviruses harboring sh-LacZ or sh-Survivin. The ensuing inducible silenced cell lines were maintained in GM2.1 without doxycycline, and experiments with these cell lines were performed in GM3 supplemented with 0.1 µg/ml of doxycycline.

### Western blot analysis

Immunoblotting methods are as described previously [41]. In full, cells were lysed at 4°C with RIPA buffer (PBS, 1% Nonidet P-40, 0.5% sodium deoxycholate, 0.1% sodium dodecyl sulfate) supplemented with 1 mM sodium orthovanadate 1 mM EDTA, 2.5 mM sodium pyrophosphate, 1 mM β-glycerophosphate, Complete Mini-EDTA-free Protease Inhibitor Mixture (Roche, Indianapolis, IN), and 1 mM phenylmethylsulfonyl fluoride. Clarified cell lysates were quantified by microtiter BCA protein assay (Pierce Scientific) for equal loading. Total cell lysates (15 to 50 µg protein) along with standard markers (Invitrogen, Novex LC 5800) were heated (85°C, 5 min) in 1× SDS sample loading buffer containing 5% 2-mercaptoethanol, loaded on 4–12% BIS-Tris gradient gels (Invitrogen gels, Cat #NP0335/NP0336) and run using NuPage SDS running buffer (NP0002) in an Invitrogen Mini-Cell (200 V, 45 min). Electrophoretic transfer (35 V, 1.5 h) to nitrocellulose membranes was conducted with Nupage transfer buffer (NP0006-1) containing methanol. Following transfer, membranes were baked (65°C, 30 min), blocked (5% non-fat dry milk-TBST, 1 h at RT), washed extensively with TBST buffer, and incubated (overnight, 4°C) with primary antibody in TBST-3% BSA. The blots were washed adequately with TBST and incubated with mouse (cat# 711-035-150) or rabbit (cat# 711-035-152) secondary antibodies in non-fat dry milk-TBST for an hour at RT. The blots were then washed three times with TBST and once with TBS, developed with ECL and imaged on Fuji X ray film (Fuji Medical 100 NIF).

### Reverse transcription-polymerase chain reactions (RT-PCR)

RT was performed as described [42]. Taq Polymerase Master Mix (Promega) was used for PCR amplification of rat Survivin, using 27 cycles of the following temperature gradients: 95°C for 15 sec, 60°C for 30 sec, and 72°C for 2 min. β-Actin, amplified as above for 17 cycles, served as an internal control. The PCR primers (Integrated DNA Technologies, Inc.) applied to detect rat Survivin expression were 5′-GAGTGACATGCCACGGCTAA-3′ (forward) and 5′-CCAGGCATGGAAACATCAAG-3′ (reverse). Quantitative (Q) PCR was performed using the Bio-Rad CFX Connect Real-Time Detection System and Invitogen SYBR Green Real-Time PCR Master Mix using the above primers and conditions.

### Transient transfection and luciferase assay

Cells were transfected using polyethylenimine method as before [43]. In brief, NRP-152 cells were plated in 12-well dishes at a density of 1×105 cells/1 ml/well in GM3 medium or 5×104 cells/well in GM2.1 and transiently transfected for 3 h with 400 ng of rat Survivin-promoter-luc constructs (Full length (FL) or truncations (Trunc #1–4)), 20 ng of CMV-Renilla, and 600 ng of empty vector per well. After 3 h of transfection, cells were washed once with 1×PBS and incubated overnight in GM3 or in GM2.1, as indicated. Cells were then treated with or without LR3-IGF-I (2 nM) in the presence or absence of various agents, and after 24 h of treatment cells were extracted with passive lysis buffer for measuring dual luciferase activity (Promega Corporation) with a ML3000 Microtiter Plate Luminometer.

### Adenovirus

Adenovirus shuttle vectors (pDC515) that direct the expression of WT-Akt1 (AdMax-Myc-Akt1WT), Active-Akt1 (AdMax-Myc-Akt1Myr), KD-Akt1 (AdMax-Myc-Akt1K179M), DN-P85 (AdMax-Myc-p85αΔSH2N), and CA-P110α (AdMax-Myc-p110αCAAX) were constructed using the AdMax system (Microbix Biosystems) and high-titer adenoviruses were prepared and titered as described previously [19], [41]. In brief, cells were plated overnight in 6-well dishes at a density of 2×105 cells/2 ml GM3/well with or without doxycycline. For adenoviral infection, cells were infected for 2 h by AdMax-cont, AdMax-Akt (WT, Active, KD), AdMax-DN-P85 (DN: Dominant negative form of PI3K), or AdMax-CA-P110α (CA: constitutively active form of PI3K), and washed once with PBS followed by addition of 2 ml of GM3. Cells were then incubated overnight for recovery and treated with TGF-β (10 ng/ml) or IGF-I (2 nM) for the indicated times. Unless mentioned, all the chemical inhibitor treatments were added 2 h prior to addition of IGF-I.

### Silencing mTOR, Rictor and Raptor in NRP-152 cells

NRP-152 cells were plated at a density of 50,000 cells/2 ml GM2.1/well in six-well plates and the next day infected with lentiviruses (MOI = 7) expressing sh-LacZ, sh-mTOR, sh-Rictor and sh-Raptor, using protamine sulfate (4 µg/mL) to facilitate infection. The viral supernatant was replaced 24 h later with GM2.1±200 nM TKDI, and 72 h later cells were harvested for Western blot and cell growth analysis. Viral titers were measured by Flow Cytometry of GFP-positive cells, interpolating the ID_50_ (viral dose for 50% infection) values for reliable quantification of viral titers.

### Cell growth assays

Unless indicated, cells were plated at a density of 5×10^3^ cells/1 ml/well in 12 well plates with GM3, and the following day treated with various indicated agents 2 h prior to addition of LR^3^-IGF-I (2 nM) or vehicle. Cell growth was assessed either enumerating single cells (following trypsinization) with a Coulter Electronics counter or by staining adherent cells in wells with crystal violet. For the latter assay, adherent cells were washed with PBS, fixed in 2% formalin/PBS (RT, 10 min) and stained with 0.2 mg/ml Crystal Violet in PBS (2 min, RT). Stained cells were washed twice with PBS, and the dye was eluted with 1% Triton/PBS (37°C, 30 min). One hundred µl of the eluted dye was transferred to a 96-well plate for assessing optical adsorption at 550 nm with a Tecan microplate spectrophotometer.

## Results

### IGF-I induces the expression of survivin

**Figure 1:**
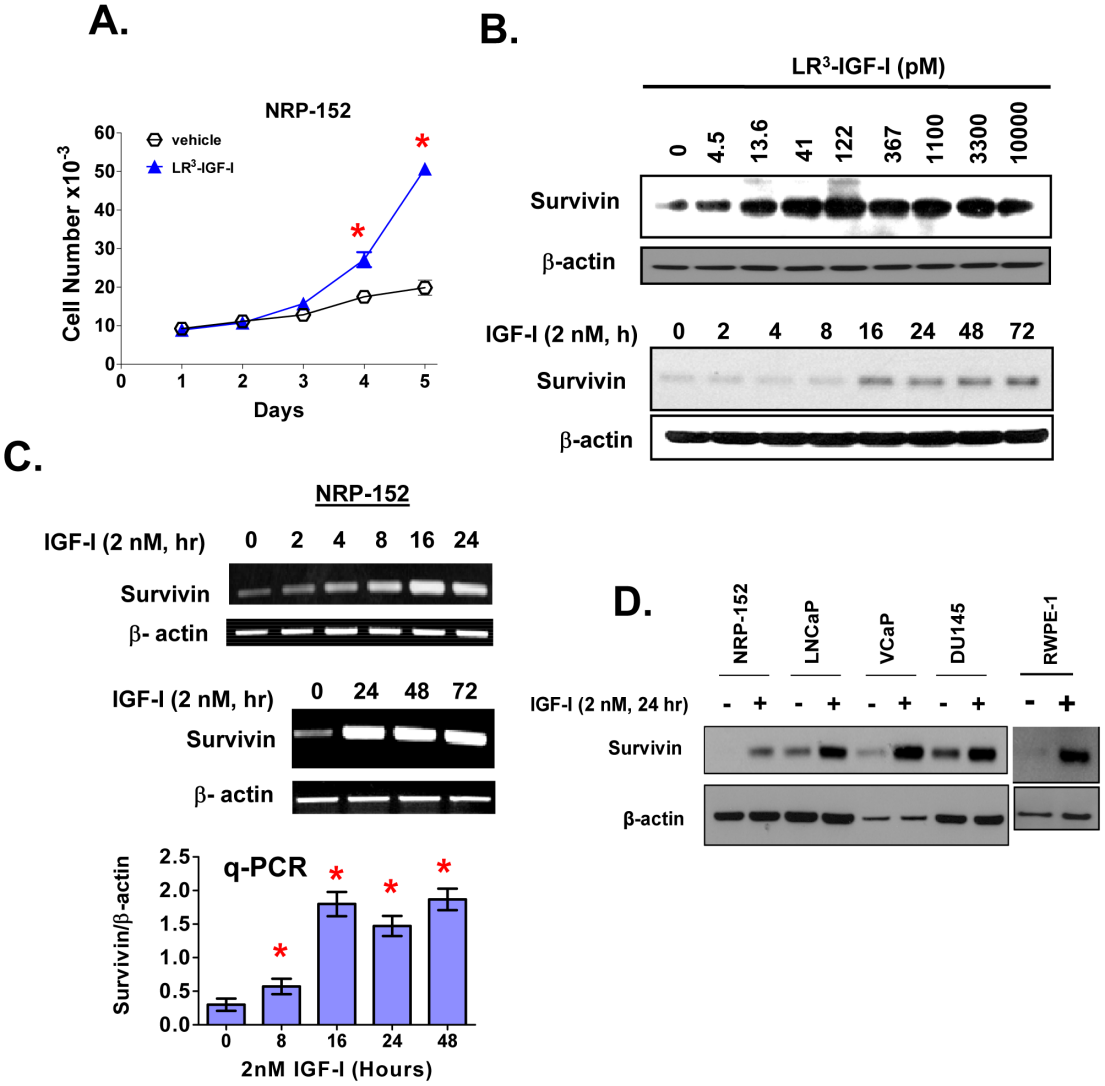
IGF-I decreases cell doubling time and induces the expression of Survivin at both mRNA and protein levels in the NRP-152 rat prostatic epithelial cell line. (A) Growth of NRP-152 cells in response to 2 nM LR3-IGF-I or vehicle in GM3 was monitored by changes in cell number daily for 5 days. (B) Effect of IGF-I on Survivin expression and activation of Smads 2 and 3 in NRP-152 cells. Cells were treated with LR3-IGF-I at the indicated concentrations for 24 h (Top) or with 2 nM of LR3-IGF-I for the indicated times from 2 to 72 h (Bottom). Survivin, P-Smad3, P-Smad2 were measured by Western blot analysis. (C) Time dependent induction of Survivin mRNA expression by 2 nM LR3-IGF-I, as demonstrated by semi-quantitative RT-PCR and real-time q-PCR. (D) IGF-I induces the expression of Survivin in malignant and non-tumorigenic prostate epithelial cell lines. Cells were cultured in DMEM/F12 medium supplemented with 1% FBS (LNCaP, VCaP, DU145), DMEM/F12 alone (RWPE-1) or GM3 (NRP-152, RWPE-1), treated with or without 2 nM LR3-IGF-I for 24 h prior to Western blot analysis. Data shown are representative of two or three independent experiments (A–D). Data in A and C are the average of triplicate determination ± S.E.; *p<0.01 by two-way Anova analysis of variance.

Survivin over-expression correlates with the aggressiveness of PCa and resistance to both chemo- and anti-androgen therapies. However, the mechanisms by which Survivin is overexpressed in cancers remain poorly understood. We previously reported that TGF-β plays a key role in maintaining low levels of Survivin in normal prostate epithelial cells, and proposed that loss of the tumor suppressor function of TGF-β significantly elevates Survivin expression in PCa. In the current study we explored the regulation of Survivin expression by the IGF-I/PI3K/Akt pathway, which has been reported to be over-activated in the majority of prostate tumors. For much of this study we used a spontaneously immortalized preneoplastic cell line (NRP-152) derived from the preneoplastic prostate of a Lobund Wistar rat [38]. NRP-152 cells require IGF-I (or a level of insulin that activates the IGF-I receptor), for growth and survival [35] through mechanisms that remain incompletely understood [40], [44], [45], [46]. To test the action of IGF-I on the IGF-I receptor (IGF-IR), we used a modified form of IGF-I, LR^3^-IGF-I, which has similar affinity for IGF-IR but binds poorly to IGF-I binding proteins [35]. The inclusion of 2 nM LR^3^-IGF-I in GM3 medium decreased the doubling time of NRP-152 cells to<24 h after a two day lag (Fig. 1A). Under these conditions, LR^3^-IGF-I induced expression of Survivin protein by 16 h (Fig. 1B), and Survivin mRNA by 8 h as demonstrated by semi-quantitative and quantitative RT-PCR (Fig. 1C), consistent with a transcriptional mechanism. Moreover, such induction occurred within a physiological range of IGF-I (5 to 10 pM) (Fig. 1B). We also showed that LR^3^-IGF-I can elevate Survivin expression in several human prostate cell lines, including the androgen dependent LNCaP and VCaP, the androgen receptor (AR)-negative DU145, and the immortalized non-tumorigenic RWPE-1 (Fig. 1D).

### Survivin expression is pivotal to cell proliferation by IGF-I

**Figure 2:**
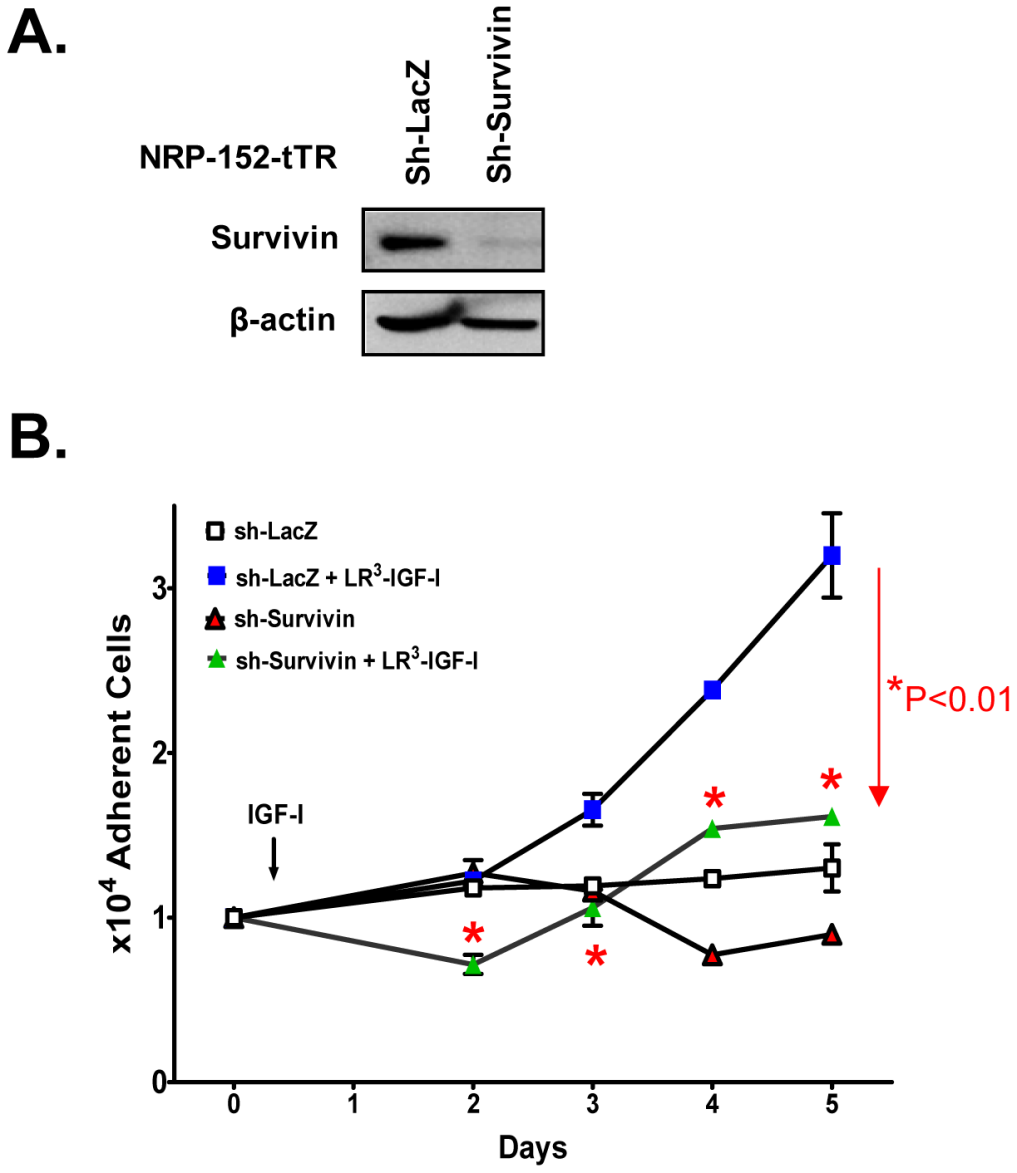
Silencing of Survivin in NRP-152 cells represses IGF-I-induced cell growth. NRP-152-tTR-sh-LacZ (control) and NRP-152-tTR-sh-Survivin cells were incubated for 48 hrs in GM3 medium with 0.1 µg/ml of doxycycline, the next day cells were treated with either vehicle or 2 nM LR^3^-IGF-I, Survivin expression was assessed after 2 days by Western blot (A) and cell growth was monitored daily using a Coulter Electronics Cell Counter (B). Data shown are the average of triplicate determinations ± S.E. Two-way Anova support that shSurvivin suppression of IGF-I-induced cell growth was statistically significant (*p<0.01).

To examine whether the induction of Survivin expression by LR3-IGF-I is necessary for its ability to promote growth of prostate epithelial cells, we stably silenced expression of Survivin in NRP-152 cells using a doxycycline-inducible shRNA lentiviral transduction system [24] (Fig. 2A). The stably silenced cells (sh-Survivin, or control LacZ-shRNA) were plated in 12-well dishes, treated with 2 nM LR3-IGF-I or vehicle, and cell growth was monitored daily for the next four days. While the basal growth rate of the sh-Survivin cells was slightly suppressed relative to that of the sh-LacZ cells, the sh-Survivin cells were refractory to growth stimulation by IGF-I compared to the marked proliferation of sh-LacZ cells by IGF-I (Fig. 2B). These results suggest that induced expression of Survivin by IGF-I is critical to proliferation of prostate epithelial cells by this mitogen.

### PI3K and Akt are critical to induction of Survivin by IGF-I

**Figure 3:**
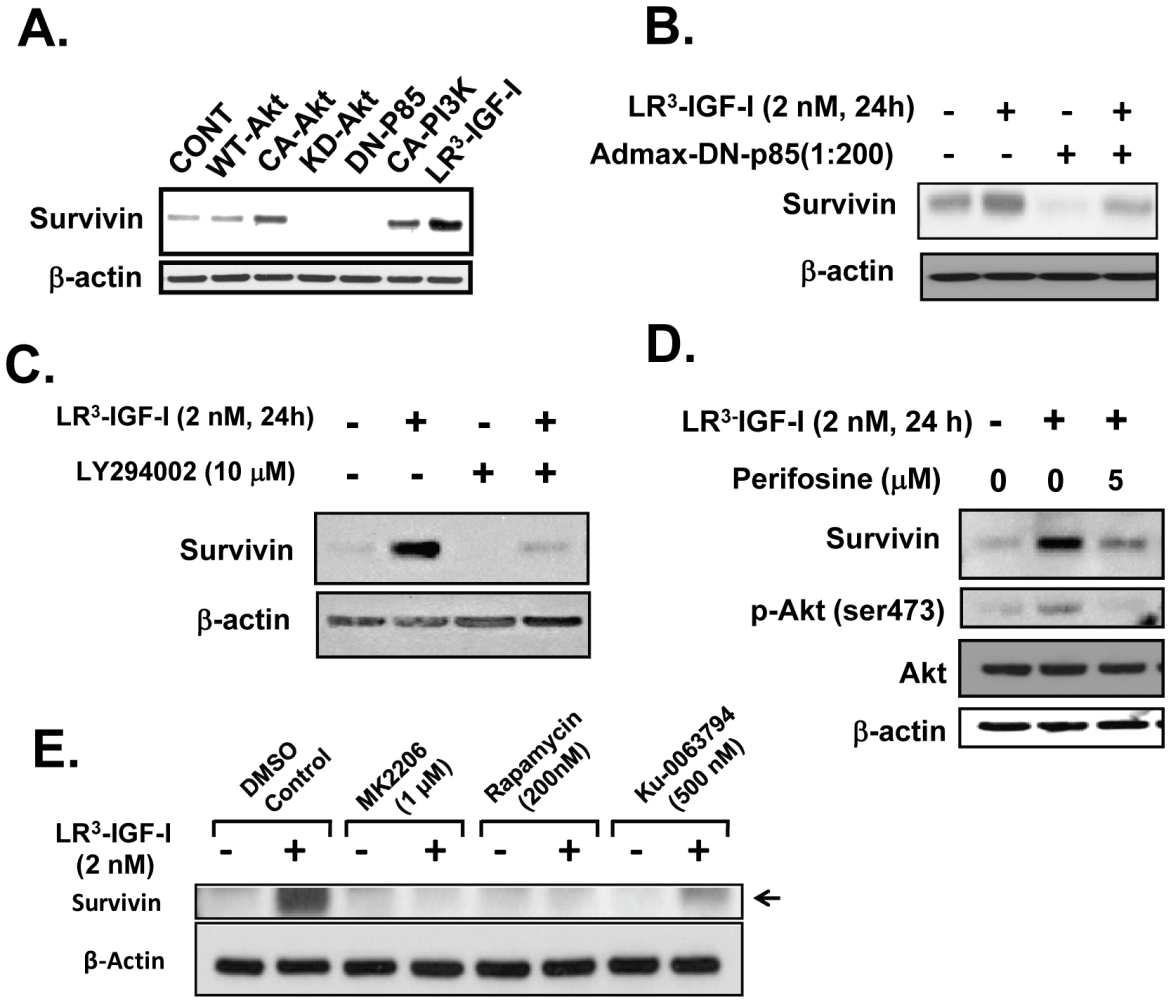
Induction of Survivin by LR^3^-IGF-I in NRP-152 cell occurs through a PI3K/Akt/mTOR pathway. (A) NRP-152 cells were infected with AdMax adenoviruses harboring WT-Akt, Myr-Akt (Act-Akt), Akt^K179M^, KD-Akt, (p85αΔSH2N), CAAX-p110α (CA-PI3K), or treated with vehicle or LR^3^-IGF-I (2 nM) for 24 h. B) NRP-152 cells were infected with AdMax KD-PI3K overnight prior to a 24 h treatment with 2 nM LR^3^-IGF-I or vehicle. NRP-152 cells were pre-treated with 10 µM LY294002 (C), 5 µM perifosine (D), 1 µM MK2206, 200 pM rapamycin or 500 nM Ku-0036794 (E) overnight prior to a 24 h treatment with 2 nM LR^3^-PI3K or vehicle. Samples were subjected to Western blotting of Survivin. Results are representative of two to three independent experiments.

We next investigated the mechanism by which IGF-I induces Survivin expression in NRP-152 cells. PI3K and Akt are activated by IGF-IR and critical to IGF-I's anti-apoptotic and proliferative responses [19]. To explore the role of these kinases in the induction of Survivin expression by LR^3^-IGF-I, NRP-152 cells were first transduced with adenoviruses carrying constitutively-active (CA) and dominant-negative (DN) PI3K and Akt. Cells then received 2 nM LR^3^-IGF-I and their Survivin levels were assessed 24 h later (Fig 3A). This experiment revealed that CA-PI3K and CA-Akt each induced Survivin expression, whereas DN-PI3K and DN-Akt suppressed basal levels of Survivin, although induction of Survivin by LR^3^-IGF-I appeared to be more robust than that induced by CA-PI3K or CA-Akt alone. While enforced expression of CA-PI3K or CA-Akt alone did not induce the expression of Survivin as robustly as by treatment with LR^3^-IGF-I, DN-PI3K repressed the induction of Survivin expression by LR^3^-IGF-I (Fig. 3B). The small chemical inhibitors of PI3K (LY294002), Akt (perifosine, MK2206) and mTOR (rapamycin, Ku-0063794) similarly repressed LR^3^-IGF-I induction of Survivin expression (Fig. 3C–D). These results implicate a role of the PI3K/Akt/mTOR pathway in IGF-I induction of Survivin expression.

### Transcriptional control of Survivin expression by IGF-I

**Figure 4:**
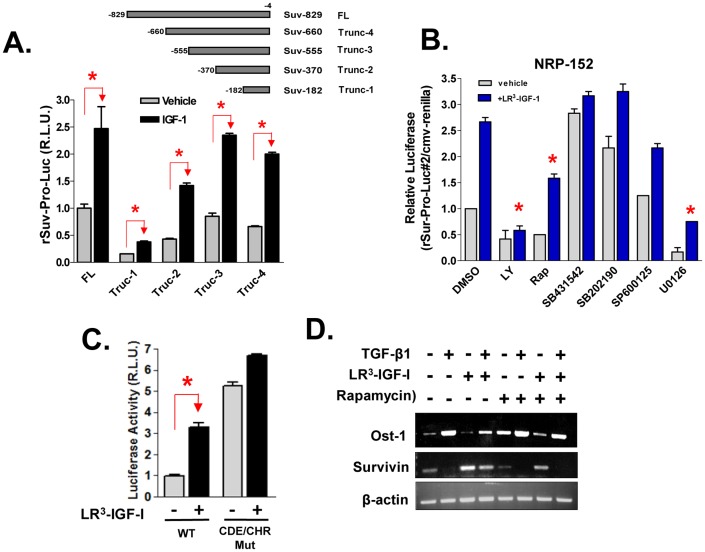
Transcriptional regulation by IGF-I of Survivin. (A) NRP-152 cells were co-transfected with full length (FL) or truncations (Trunc-1 to -4) of rat Survivin promoter-Firefly luciferase reporter constructs, along with CMV-Renilla control reporter one day before a 24 h treatment with LR^3^-IGF-I or vehicle, and cells were then analyzed for dual luciferase reporter activity. (B) NRP-152 cells were co-transfected with Trunc-2 Survivin promoter-luciferase construct (rSur-pro-Luc#2) and CMV-Renilla as in A, and next day cells were treated with various kinase inhibitors (LY: 10 µM LY294004; Rap: 200 pM rapamycin; 10 µM of either SB431542, SB202190, SP600125 or U0126) or DMSO vehicle for 2 h before 24 h treatment with 2 nM LR^3^-IGF-I or vehicle. (C) NRP-152 cells were co-transfected with rSur-pro-Luc#2 (WT) or rSur-pro-Luc#2 mutated at CDE and CHR (CDE/CHR Mut) along with CMV-Renilla, next day cells were treated with 2 nM LR^3^-IGF-I or vehicle, and harvested for dual luciferase activity. Data shown are relative values of Firefly luciferase normalized to Renilla luciferase, and expressed as relative luciferase units (R.L.U.). Each bar represents the average of triplicate determinations ± S.E. Statistical significance (*p<0.01) was assessed by two-way Anova analysis of variance. D) IGF-I reverses the ability of TGF-β to suppress Survivin mRNA and such reversal is mitigated by rapamycin. NRP-152 cells plated in GM3 overnight were pre-treated for 2 h with either 200 nM rapamycin or vehicle, followed by overnight with 2 nM LR^3^-IGF-I or vehicle, and then treated with 10 ng/ml rhTGF-β1 for 24 h. RNA was extracted and processed for RT-PCR of Survivin, Osteopontin (Ost-1) and β-actin.

To examine whether IGF-I induces the expression of Survivin through a transcriptional mechanism (as suggested in Fig. 1C), NRP-152 cells were transfected with constructs of the rat Survivin promoter fused to a Firefly luciferase reporter along with a CMV-Renilla luciferase reporter (to control for transfection efficiency) (Fig. 4A). The next day, cells were treated with 2 nM LR^3^-IGF-I and after 24 h Firefly luciferase activity was measured and normalized to Renilla luciferase. While the smallest construct of the Survivin promoter used (182 bp) gave the lowest basal activity, it conferred a similar fold induction by LR^3^-IGF-I relative to the other promoter constructs (Fig. 4A). These results suggest that the IGF-I-dependent responsive element(s) reside within the minimal promoter construct (−182/−4), supporting our hypothesis that IGF-I induces Survivin expression by suppressing the activation of the pocket proteins.

We next assessed the effect of various small chemical inhibitors on the ability of IGF-I to activate the Survivin promoter using the second smallest (−370/−4) construct. The PI3K inhibitor LY294002 effectively and fully repressed basal and IGF-I-induced activity of the Survivin promoter, respectively (Fig. 4B). Rapamycin and the mitogen-activated kinase (MAPK) kinase (MEK) inhibitor U0126 effectively repressed basal promoter activity, and partially inhibited promoter activation by LR^3^-IGF-I. Interestingly, the TβRI kinase inhibitor SB431542 substantially induced the expression of Survivin to the level induced by LR^3^-IGF-I, and combined treatment with LR^3^-IGF-I did not further enhance promoter activity. The p38 MAPK inhibitor SB202190 partially induced the activity of that Survivin promoter construct and blunted the overall induction by LR^3^-IGF-I, whereas the c-Jun N-terminal kinase (JNK) inhibitor SP600125 partially blunted promoter activation by LR^3^-IGF-I. As SB202190 partially antagonizes the TβRI kinase, it is likely that activation of this promoter by SB202190 is primarily through inactivation of TβRI. These results suggest that IGF-I induces Survivin expression principally by blocking endogenous TGF-β.

Previous work showed Rb or other pocket proteins (p107, p130) in association with E2F4 bind to CDE and CHR response elements of the Survivin promoter and repress promoter activity [24], [47], and we previously reported that TGF-β down-regulates the Survivin promoter through activating the pocket proteins [24]. The effect of IGF-I on induction of a Survivin promoter construct (−370/−4) with mutant CHR and CDE response elements (within the proximal promoter region) was thus investigated. Point mutations in both the CHR and CDE sites induced promoter activity and blunted the response to LR^3^-IGF-I (Fig. 4C), suggesting that most of the induction of Survivin by IGF-I requires CHR and CDE, the same elements required for suppression of the Survivin promoter by TGF-β. Consistent with this possibility, we showed LR^3^-IGF-I at least partially reversed the suppression of Survivin mRNA expression by TGF-β, whereas rapamycin reversed the protection by LR^3^-IGF-I and significantly repressed Survivin induction by LR^3^-IGF-I (Fig. 4D). The mRNA levels for the secreted glycosylated phosphoprotein osteopontin (Ost-1) exhibited the opposite pattern of regulation, as LR^3^-IGF-I repressed Ost-1 induction by TGF-β and rapamycin reversed this IGF-I repression.

### IGF-I represses the Survivin promoter through inhibiting TGF-β receptor signaling

**Figure 5:**
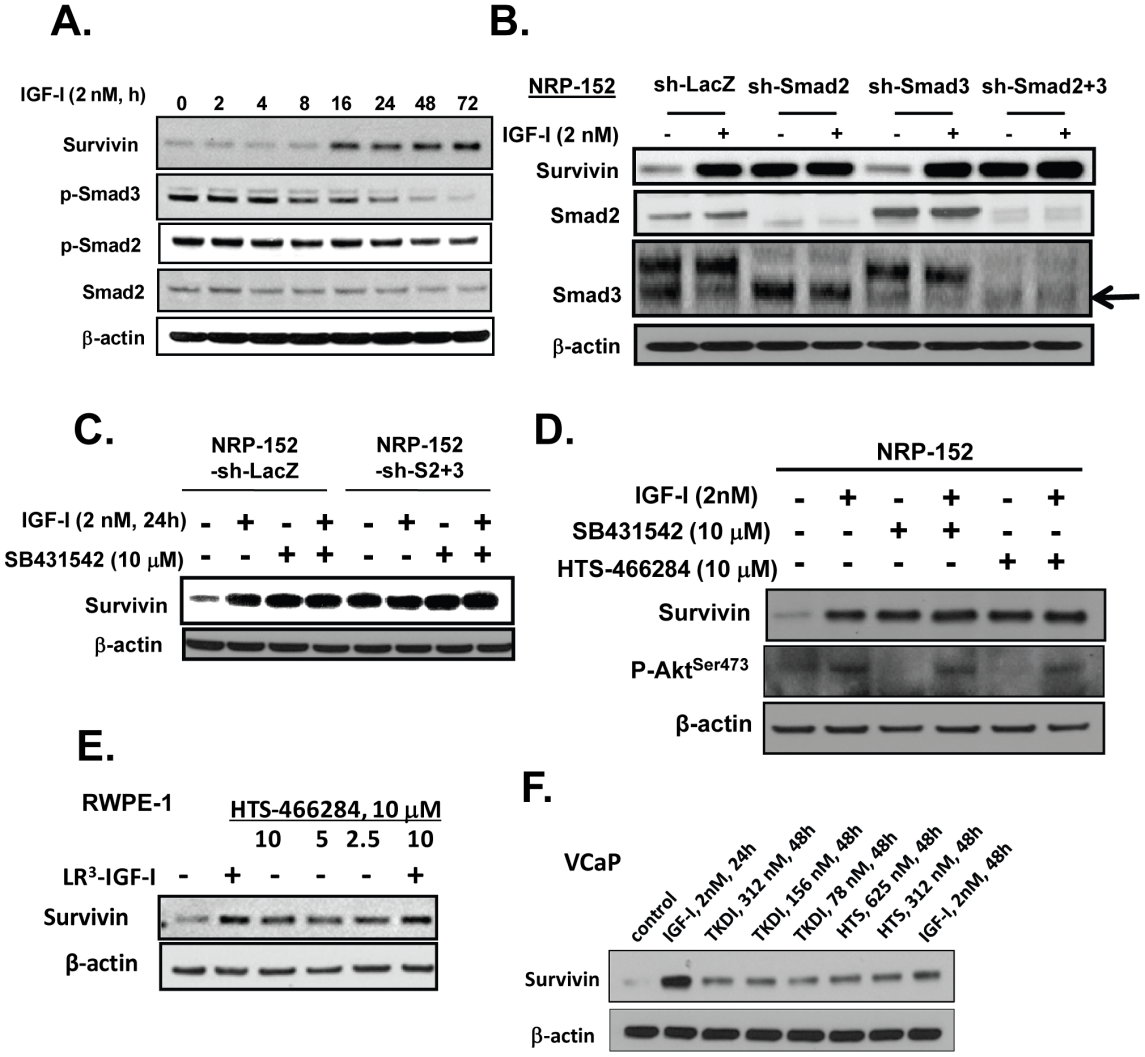
IGF-I induces Survivin through a Smad dependent mechanism. (A) NRP-152 cells plated overnight in GM3 were treated at various times with LR^3^-IGF-I for up to 72 h, and cell lysates were analyzed for Western blot expression of Survivin, and P-Smads 2 and 3. (B) NRP-152 cells stably expressing sh-Smads 2, 3, and 2+3 or lentiviral sh-LacZ (control) were treated with 2 nM LR^3^-IGF-I or vehicle for 24 h prior to Western blot analysis for Survivin and Smads 2 and 3. (C) NRP-152 cells stably expressing sh-Smads 2+3 or lentiviral sh-LacZ (control) were treated with DMSO vehicle or 10 µM SB431542 for 2 h prior to treatment with 2 nM LR^3^-IGF-I or vehicle for 24 h, and changes in Survivin expression was assessed by Western blot analysis. (D) NRP-152 cells were treated with either 10 µM HTS466284 or 10 µM SB43152 for 2 h prior to treatment with 2 nM LR^3^-IGF-I or vehicle for 24 h, and changes in Survivin expression were assessed by Western blot analysis. (E,F) RWPE-1 and VCaP cells plated in GM3 were treated with LR^3^-IGF-I and the TGF-β receptor kinase inhibitors HTS466284 (HTS) or TKDI for 24 h prior to lysing cells for Western blot analysis of Survivin expression. Results are representative of two to three separate experiments.

Previous studies from our group indicated that IGF-I suppresses the ability of TGF-β to activate Smad3 [40], [45]. We now show that LR3-IGF-I suppresses the levels of endogenous phospho(P)-Smad3 (and to a lesser extent P-Smad2) in a time-dependent manner that matches the induction of Survivin protein by LR3-IGF-I (Fig. 5A). To test whether IGF-I's ability to inhibit Survivin induction occurred through suppression of Smad activity, we used NRP-152 cells that were stably silenced for the expression of Smads 2 or/and 3 by shRNA lentiviral transduction [31]. Cells were treated with either 2 nM LR3-IGF-I or vehicle, and the expression of Survivin was assessed 24 h later by Western blotting (Fig. 5B). Cells stably expressing sh-Smad2 or sh-Smad2+3, but not sh-Smad3 alone expressed enhanced levels of Survivin relative to control (sh-LacZ). Treatment with LR3-IGF-I induced Survivin expression in sh-LacZ and sh-Smad3 cells, similar to that induced without LR3-IGF-I in sh-Smad2 cells. Moreover, levels of Survivin were not further enhanced in sh-Smad2 or sh-Smad2+3 cells treated with LR3-IGF-I relative to vehicle (Fig. 5B), and suppression of TGF-β receptor signaling with a TβRI kinase inhibitor, SB431542 (10 µM), which alone induced Survivin expression to levels similar to that induced by LR3-IGF-I in sh-LacZ cells, did not further induce Survivin expression when combined with LR3-IGF-I in sh-LacZ cells or with sh-Smad2+3 (Fig. 5C). Treatment of parental NRP-152 cells with SB431542 (10 µM) or another TβRI inhibitor, HTS-466284 (10 µM), each induced Survivin expression to the same level as that induced by 2 nM LR3-IGF-I alone, and combined treatments with these agents did not further enhance Survivin levels. Together these data strongly suggest that all effects of LR3-IGF-I on inducing levels of Survivin in NRP-152 cells occurs through reversing TGF-β autocrine activity. The above TβRI kinase and another more specific TβRI Kinase Domain Inhibitor (TKDI: 2-(3-(6-Methylpyridin-2-yl)-1H-pyrazol-4-yl)-1,5-naphthyridine) [48] also induced Survivin levels in RWPE-1 and VCaP cells, but did not further enhance the induction of Survivin by IGF-I alone (Fig. 5E,F and data not shown).

### IGF-I stimulates cell growth through reversing growth suppression by endogenous TGF-β

**Figure 6:**
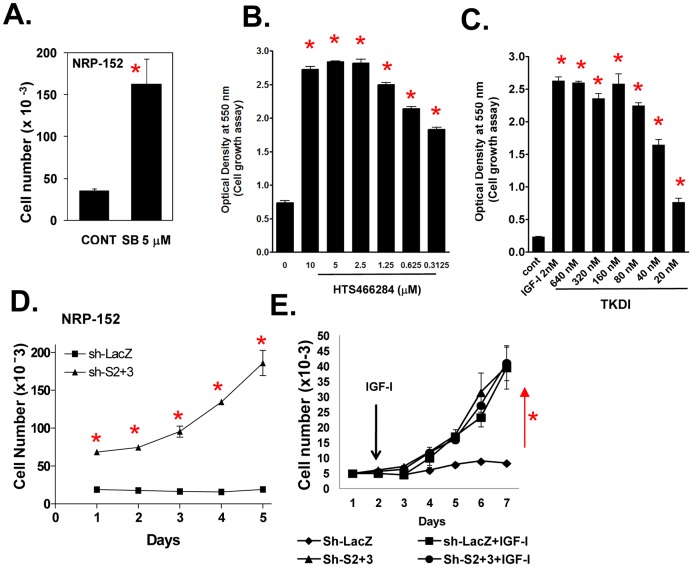
IGF-I enhances cell growth by suppressing TGF-β autocrine signaling. (A–C) TGF-β receptor kinase inhibitors stimulate the growth of NRP-152 cells. NRP-152 cells plated were treated with 5 µM of SB431542 (SB) (A) or with various concentrations of HTS466284 or TKDI and cell growth was measured 6 days later by counting cell number (A) or by crystal violet staining of fixed cells (B,C). (D) Growth of NRP-152-Sh-Smad2+3 cells versus NRP-152-Sh-LacZ cells in GM3 medium. (E) NRP-152-sh-LacZ and NRP-152-sh-Smad2+3 cell lines were incubated in the presence or absence of LR^3^-IGF-I (2 nM) for 5 days and cell growth was monitored daily for 5 days (D,E). Percent of growth inhibition by rapamycin in NRP-152-sh-LacZ and NRP-152-sh-Smad2+3 cell lines. Cell numbers were measured using Coulter Electronics Counter. Data shown are the average of triplicate determinations ± S.E. (*p<0.01). Statistical significance (*p<0.01) was assessed by two-way Anova analysis of variance.

We next examined whether the ability of IGF-I to stimulate growth of NRP-152 cells was through suppressing autocrine activity of TGF-β. For this, NRP-152 cells were plated overnight in GM3 medium, treated with various TβRI kinase inhibitors and changes in cell growth was assessed after 5 to 6 days by counting total cell numbers and by crystal violet staining of fixed cells. Each of these TβRI kinase inhibitors enhanced cell growth between 4- to 10-fold (Fig. 6A–C). The most active and specific of these inhibitors, TKDI, optimally induced growth of NRP-152 cells (in GM3 medium) to the same level as that by LR3-IGF-I, indicating that both activation of IGF-IR and selective suppression of the TβRI kinase are equally effective in promoting the growth of NRP-152 cells under the same condition. TKDI maximally inhibits TGF-β receptor signaling at 0.1 to 0.2 µM, whereas ≤16 µM TKDI had minimal effects on 9 closely related kinases, including p38-MAPK [48]. To examine the role of Smads 2 and 3 as mediators of this growth response, we compared 5-day growth rates of sh-Smad2+3 NRP-152 versus sh-LacZ NRP-152 in GM3 medium. Relative to control (sh-LacZ), silencing Smads 2 and 3 stimulated robust cell proliferation (Fig. 6D). In another experiment, daily changes in growth of sh-LacZ and sh-Smad2+3 cells was assessed each in the presence and absence of 2 nM LR3-IGF-I for 6 days (Fig. 6E). LR3-IGF-I induced growth of sh-LacZ cells similar to that of the sh-Smad2+3 cells without LR3-IGF-I, and addition of LR3-IGF-I did not further promote the growth of the shSmad2+3 cells. These results indicate that the mitogenic activity of LR3-IGF-I and of silencing Smad2+3 are essentially the same, and suggest that the effects of IGF-I on growth of NRP-152 cells are entirely through repressing the growth inhibitory activity of autocrine TGF-β, which is dependent on the activation of Smad2+3, similar to the regulation of Survivin expression by TGF-β [24].

### Role of TGF-β signaling as a mediator of growth suppression and inhibition of Survivin expression by inhibitors of PI3K, Akt, mTOR and MEK

**Figure 7:**
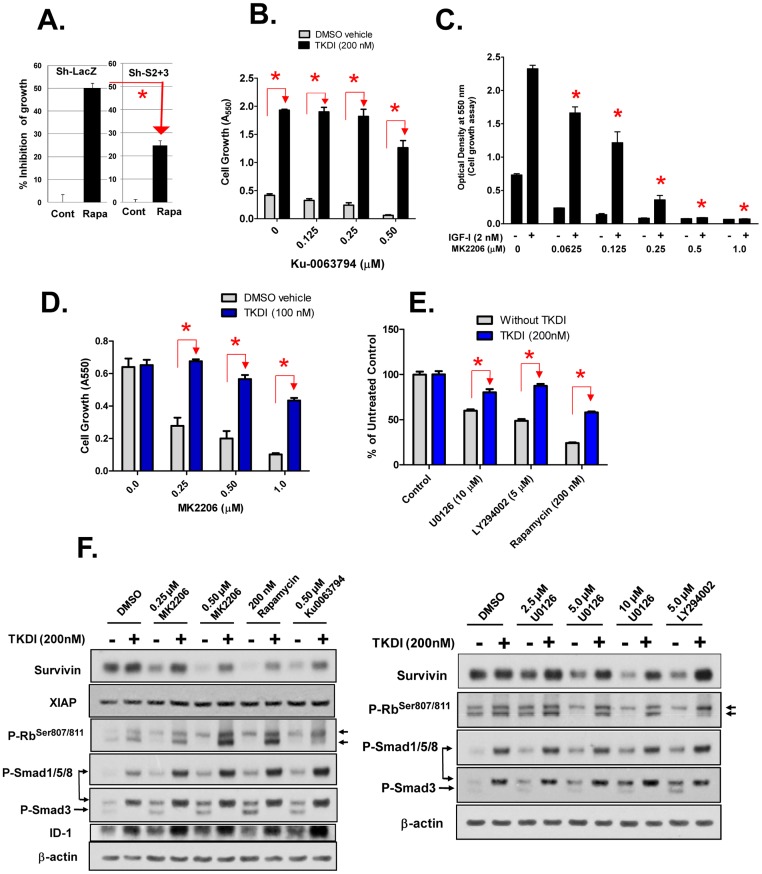
Role of TGF-β signaling in suppression of cell growth, Survivin expression and activation of Rb by antagonists of mTOR, Akt, PI3K and MEK in prostate epithelial cells. The ability of the mTORC1 inhbitor rapamycin (A) and the mTORC1 and mTORC2 dual inhibitor Ku-0063794 (B) to inhibit growth of NRP-152 cells is revered by silencing Smads 2 and 3 (sh-S2+3) or by the TβRI/TβRII kinase dual inhibitor (TKDI), as assessed by cell growth after 6 days in GM3 medium. Although the Akt inhibitor MK2206 (0.5 µM) effectively represses LR3-IGF-I-induced cell growth in GM3 (C), TKDI reverses such suppression (D) as well as growth suppression (E) and Survivin suppression (F) by LY294002, rapamycin, U0126, MK2206 and Ku-0063794. D,E,F) Cells were plated in GM2.1 and pre-treated with TKDI or vehicle 4 h prior to treatment with the other kinase inhibitors. Cells were examined for cell growth after 5 days (by crystal violet staining) (D,E) or for expression of Survivin, XIAP, P-Smad3, P-Smad1/5/8, P-Rb, and ID-1 (by Western blot) after 24 h treatment (F). Data shown are the average of triplicate determinations ± S.E; Statistical significance (*p<0.01) was determined by two-way Anova.

The above results support our hypothesis that IGF-I promotes the growth of NRP-152 cells and their expression of Survivin through inactivating autocrine TGF-β/Smad activity. We next explored the impact of the signaling pathway activated by IGF-I on cell growth and Survivin expression by autocrine TGF-β. When cultured in GM3, NRP-152 cells undergo increased cell death/growth arrest by rapamycin (Fig. 7A)[40]. This activity of rapamycin was significantly reduced in sh-Smad2+3 versus sh-LacZ NRP-152 cells, suggesting that the growth suppressive activity of mTORC1 suppression is partly dependent on expression of Smads 2 and/or 3. In a similar experiment, we showed that suppression of growth by the mTORC1+2 kinase inhibitor, Ku-0063794, was effectively blocked by pre-treatment with 200 nM TKDI (Fig. 7B). In Fig. 7C we show that 0.25 to 1.0 µM of the Akt kinase inhibitor MK2206 effectively blocked the ability of LR3-IGF-I to promote growth of NRP-152 cell. MK2206 also effectively represses growth of NPR-152 cells under optimal growth conditions (in GM2.1) (Fig. 7D). Of note, GM2.1 contains a level of insulin (5 µg/ml) that engages IGF-IR; previous studies demonstrated that insulin (or IGF-I) is essential for logarithmic growth of NRP-152 cells [49]. Under those conditions, TKDI did not enhance cell growth; however, it effectively reversed the cytostatic activity of MK2206 (Fig. 7D). TKDI similarly reversed the cytostatic activity of 10 µM U0126, 5 µM LY294002 or 200 nM rapamycin (Fig. 7E). Moreover, each of the above kinase inhibitors within 24 h suppressed Survivin at the protein and promoter level, and such suppression was reversed by pre-treatment with TKDI (Fig. 7F, 8A). In contrast, levels of a structurally related protein (XIAP) were not altered by inhibition of mTOR, Akt or TGF-β (Fig. 7F). Similar changes in Ser807/811 phosphorylation of Rb (Fig. 7F), consistent with the role of TGF-β in the activation of Rb and our previous report that inactivation of Rb and Rb-like proteins regulate activity of the Survivin promoter [24]. Using a P-Smad3Ser423/425 antibody, we found that each of those inhibitors also activated P-Smad3 (lower band) and P-Smad1/5/8 (upper band), the latter of which was confirmed with a P-Smad1/5/8 selective antibody. As expected, TKDI inhibited P-Smad3 but not P-Smad1/5/8. Interestingly, TKDI instead robustly enhanced P-Smad1/5/8 levels, which were further enhanced by mTOR and Akt inhibitors. ID-1, a transcriptional target of Smads 1, 5 and 8, was also induced in parallel with P-Smad1/5/8. Together, these results suggest that the cytostatic activities of inhibitors of PI3K, Akt, mTOR or MEK, which also reduced Survivin expression, are largely dependent on an autocrine TGF-β signaling pathway.

**Figure 8:**
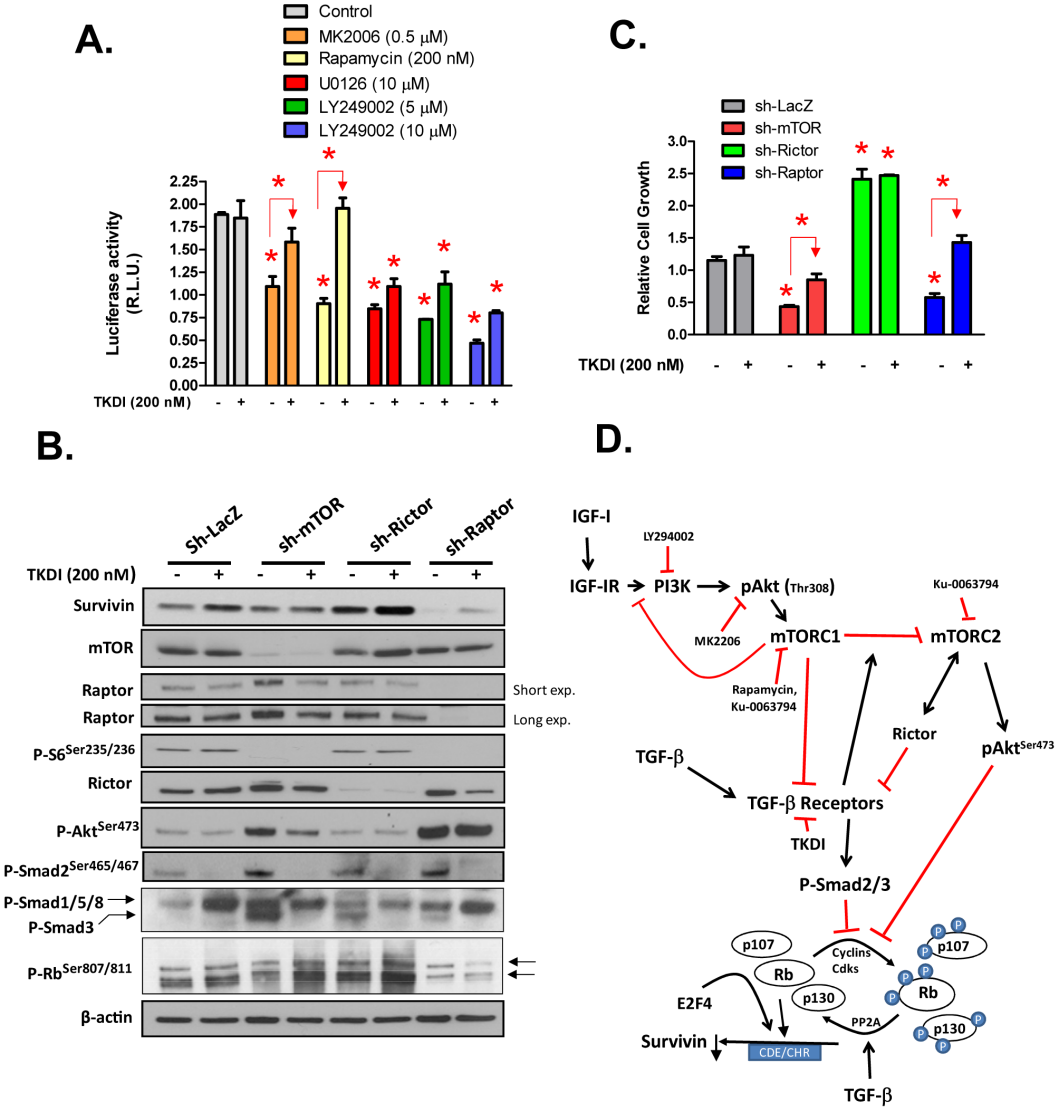
Role of mTORC1 and mTORC2 in control of Survivin suppression and Rb activation by autocrine TGF-β. (A) Inhibition of autocrine TGF-β signaling reverses suppression of the Survivin promoter by antagonists of PI3K, Akt, mTOR or MEK. NRP-152 cells were transiently transfected with WT rSur-pro-Luc#2 promoter reporter construct, cultured overnight in GM2.1, and treated with 200 nM TKDI for 4 h prior to the indicated kinase inhibitors; dual luciferase activity was measured 24 h later. (B,C) NRP152 cells were plated overnight at a density of 50,000 cells/well/2 ml GM2.1 in six-well dishes, and were infected lentiviruses expressing with sh-LacZ, sh-mTOR, sh-Rictor and sh-Raptor for 24 h. Viral supernatants were replaced with GM2.1, cells were treated with 200 nM TKDI for 72 h, and cells then were harvested for Western blotting analysis of mTOR, Rictor, Raptor, Survivin, P-Rb^807/811^, P-Akt^Ser473^, P-S6^Ser235/236^ and P-Smad2^Ser465^/467 (B) and cell growth by a BCA protein assay (C). Error bars represent S.E. or triplicate determinations, and statistical significance (*p<0.01) was assessed by two-way Anova. Schematic model depicting a central role of TGF-β as regulator/mediator of Survivin expression by IGF-I/PI3K/Akt/mTOR signaling.

### Differential roles of Raptor, Rictor and mTOR in regulating expression of Survivin

mTOR resides in two functionally unique complexes: mTORC1 and mTORC2. mTORC1 is the rapamycin sensitive complex that is distinguished from mTORC2 by the presence of Raptor and the ability to phosphorylate p70-S6K; and mTORC2 is distinguished from mTORC1 by the presence of Rictor and the unique ability to phosphorylate Akt at serine 473 [50]. We studied the selective role of mTORC1 versus mTORC2 in TGF-β-dependent regulation of Survivin expression and cell growth, by individually silencing the expression of mTOR, Raptor and Rictor in NRP-152 cells with shRNA lentiviral-mediated transduction. Virally transduced cells were cultured in GM2.1 in the presence of either 200 nM TKDI or DMSO vehicle for 3 days, and alterations in the expression of Survivin, the activity of mTORC1 (by P-S6^Ser235/236^), mTORC2 (by P-Akt^Ser473^), Smad2 (P-Smad2^Ser465/467^), Smad3 (P-Smad3^Ser423/425^) and Rb (P-Rb^Ser807/811^) were assessed by Western blot analysis (Fig. 8B), and compared to changes in cell growth (Fig. 8C). Relative to sh-LacZ, Survivin expression was repressed by sh-Raptor, elevated by sh-Rictor, but not altered by sh-mTOR. Interestingly, TKDI elevated Survivin expression in sh-LacZ, sh-Rictor and sh-Raptor cells, but not in sh-mTOR cells. As expected, silencing either mTOR or Raptor but not Rictor significantly repressed P-S6^Ser235/236^. Unexpectedly, however, silencing Rictor did not repress P-Akt^Ser473^ levels, while silencing mTOR or Raptor each enhanced P-Akt^Ser473^, suggesting that mTORC2 is typically inactive in those cells where it is robustly suppressed by mTORC1. This further suggests that elevation of Survivin expression by sh-Rictor occurs independently of the disruption of mTORC2 complex (Fig. 8D).

Despite their differential effects on the regulation of Survivin expression, sh-mTOR, sh-Raptor and sh-Rictor (compared with sh-LacZ) each activated TβRI (as assessed by elevation of P-Smad2^Ser465/467^ and P-Smad3^Ser423/425^; note that TKDI ablated P-Smad2^Ser465/467^ and P-Smad3^Ser423/425^), indicating that mTORC1 represses or/and mTORC2 activates TGF-β signaling; and this also opens up the likelihood that an mTORC2-independent function of Rictor represses Smad activation. The anti-P-Smad3^Ser423/425^ IgG used recognizes P-Smad3 (lower band, blocked by TKDI) as well as P-Smad1/5/8 (upperband, not blocked by TDKI). TKDI, treatment or silencing mTOR, Rictor, and Raptor each alone enhanced P-Smad1/5/8, with silencing of Rictor blunting the induction by TKDI.

TKDI enhanced P-Rb^Ser807/811^ levels in sh-mTOR and sh-Rictor cells, but not in sh-Raptor cells in which intensities of P-Rb^Ser807/811^ were significantly diminished. This suggests that either suppression of mTORC1 or/and activation of mTORC2 inhibits Survivin expression through TGF-β-dependent Rb activation, and that silencing Rictor elevates Survivin through inhibiting Rb independent of TβRI. Relative changes in cell growth were roughly an integration of increased levels of Survivin expression and suppression of P-Smads 2 and 3, with growth stimulation by sh-Rictor overriding growth suppression by P-Smad2/3 (Fig. 8C,D).

## Discussion and Conclusion

Here we provide the first evidence of a TGF-β/Survivin/mTOR axis that is critical for the ability of IGF-I to induce growth of prostate epithelial cells, using NRP-152 as a unique system. The derivation of NRP-152 line from a pre-neoplastic prostate, as well as its non-tumorigenic phenotype [38], stem cell-like features [51] and unique ability to reconstitute a functional prostate epithelium *in vivo* [52] offers an ideal model to study early stages of prostate tumorigenesis. Disruption of TGF-β receptor or Smad signaling promotes the malignant transformation of NRP-152 cells, as demonstrated by tumor growth in athymic mice [29], [31]. In our current paradigm (Fig 8D), IGF-I-induced cell growth is mediated through the neutralization of autocrine TGF-β activity, wherein IGF-I suppresses Smad2/3-dependent TGF-β signaling predominantly through an mTORC1-dependent mechanism. The resulting suppression of TGF-β signaling inactivates the Rb pocket proteins that then relieve suppression of the Survivin promoter through displacement of Rb/E2F4 from CDE/CHR response elements [24]. Consistent with Survivin's role in cell cycle progression and inhibition of apoptosis [53], the ensuing elevation of Survivin enables enhanced cell growth by IGF-I (Fig. 2). A previous study also reported that IGF-I induces the expression of Survivin, although through a different mechanism involving increased translation of Survivin mRNA rather than changes in levels of Survivin mRNA or Survivin protein stability, and occurred through mTOR-dependent activation of p70-S6K [17].

Recent work with the FET colon adenoma cell line illustrates another means (in certain cells) by which TGF-β may induce loss of Survivin expression, namely through a proteosomal mechanism involving Smad3-dependent activation of protein kinase A (PKA), which phosphorylates Survivin at Ser^20^ [54]. This phosphorylation event also promotes proteosomal degradation of XIAP, an IAP stabilized by its association with Survivin [4]. Chowdhury et al. [54] proposed that TGF-β-promotes the degradation of XIAP by an additional mechanism involving PKA-dependent activation of the phosphatase PP2A, which reverses the stabilization of XIAP by Akt-dependent phosphorylation of XIAP at Ser^87^ [55]. The involvement of PKA as an additional route by which TGF-β down-regulates Survivin expression remains to be observed in prostate epithelial cells, although our data (Fig. 5D) do not support that autocrine TGF-β inactivates Akt. Moreover, we showed that TGF-β does not down-regulate XIAP in NRP-154 and NRP-152 prostate epithelial cells ([24], Fig. 7F). However, PKA-dependent activation of PP2A may be involved in the mechanism by which TGF-β represses the Survivin promoter through the Rb pocket proteins, which are substrates PP2A homoenzymes [56].

Intriguingly, we show that suppression of TGF-β signaling by a highly-specific TGF-β receptor kinase inhibitor (TKDI)[48] can effectively reverse the suppression of growth and Survivin expression in NRP-152 cells by selective antagonists of PI3K, Akt, mTOR or MEK (Fig. 7 & 8). These data implicate that PI3K, Akt, mTORC1 and MEK each promote growth and Survivin expression by antagonizing autocrine/paracrine TGF-β signaling, albeit likely through different mechanisms. Case in point, TKDI more effectively reversed the ability of U0126 or LY294002 than rapamycin or MK2206 to suppress Survivin expression at the protein level (Fig. 7F); however, TKDI more effectively reversed the ability of rapamycin or MK2206 than U0126 or LY294006 to inhibit the Survivin gene promoter (Fig. 8A). As PI3K, Akt, mTOR and MEK are activated by numerous receptor tyrosine kinases, TGF-β could be viewed as a down-stream brake that represses growth signals commonly triggered by growth factor receptors in normal or pre-neoplastic cells. Loss of this TGF-β brake in cancer may thus lead to an exaggerated/amplified growth response and Survivin expression by otherwise homeostatic levels of IGF-IR signaling. Through the underlying mechanism, inhibition of this TGF-β break by one mitogen (i.e., IGF-I) or by loss of a key tumor suppressor, PTEN, would enhance mitogenic signaling by another mitogen. By the same token, our findings support that deregulation of TGF-β signaling in cancer or during tumor progression is likely to significantly impact the efficacy of therapeutic strategies involving inhibitors of PI3K, Akt, MEK or mTOR.

Functional loss of PTEN, which is a hallmark of most prostate cancers, robustly contributes to cell survival through the PI3K/Akt/mTOR pathway [57], [58], a pathway which is also activated by IGF-I in PCa [19], [59], [60]. Prostate targeted PTEN knockout leads to enhanced expression of Survivin through activating the Survivin promoter via reduced promoter binding of FOXO1 and FOXO3a [61], which are retained in the cytoplasm following phosphorylation by Akt [62]. A recent study revealed that while PTEN null prostates of conditional knockout mice develop tumors, their TGF-β and BMP Smads were unexpectedly activated or induced through unexplored mechanisms [34]. Based on our findings, elevated Akt/mTOR signaling in the PTEN null mice would be expected to instead abrogate activation of Smads. It is thus likely that PTEN loss activates a pathway independent of Akt signaling that leads to the activation of Smads, thus overriding the suppression of Smads by Akt/mTOR. Alternatively, Akt, which has been previously shown to bind to Smads 2 and 3 and prevent the transcriptional activity of Smad3 [40], may reverse the ability of Smads to inhibit Survivin expression in those mice.

Another fascinating observation is that TKDI, sh-mTOR and sh-Raptor but not sh-Rictor increased levels of P-Smad1/5/8 (Fig. 7F & 8B). This suggests that TGF-β signaling typically represses the activation of BMP Smads, and that loss of TGF-β signaling in cancer conversely activates the BMP signaling pathway. The molecular mechanism behind suppression of BMP signaling by TGF-β is under investigation in our group. Our results that sh-mTOR and sh-Raptor activate Smad1/5/8 are consistent with our recent article demonstrating that mTORC1 kinase represses P-Smad1/5, whereas mTORC2 activates P-Smad1/5 in human PCa cell lines [63]. Despite the activation of BMP Smad signaling, Survivin levels remain elevated. It is thus likely that suppression of autocrine TGF-β signaling may override the cytostatic effects of autocrine BMP signaling while enhancing their growth promoting effects. These potential connections and their mechanistic bases remain to be explored. Intriguingly, Raptor and Rictor levels were elevated in sh-mTOR cells (in the absence of TKDI) relative to sh-LacZ cells, and TKDI suppressed expression of both Raptor and Rictor in sh-mTOR expressing cells and suppressed expression of Rictor in sh-Raptor cells (Fig. 8B), suggesting a role for autocrine TGF-β in inducing the levels of Raptor and Rictor following loss of mTOR (Fig. 8D). Moreover, TKDI repressed the elevation of P-Akt^Ser473^ by sh-TOR but not by sh-Raptor (Fig. 8B), suggesting that increased autocrine TGF-β activity is involved in the formation of mTORC2 upon loss of mTOR but not upon loss of Raptor (Fig. 8D). Exploring the mechanistic basis behind these effects may yield better insight on alterations underlying the tumor suppressor function of TGF-β.

In summary, we provide the first evidence using a pre-neoplastic model of prostate cancer that an autocrine TGF-β loop serves as a critical barrier between the IGF-I/PI3K/Akt/mTORC1 signaling network and the induction of cell growth/survival associated with inactivation of the Rb pocket protein and induction of Survivin. As such, functional inactivation of TGF-β signaling, particularly loss of TGF-β-induced apoptosis or growth arrest, which is a common occurrence during prostate carcinogenesis, serves as a driver of malignant transformation through inactivation of Rb and induction of Survivin. As we and others have demonstrated that activation of the AR can directly antagonize TGF-β signaling [41], [43], [64], deregulated TGF-β signaling by the over-activation/dysregulation of AR signaling may mediate the resistance of castrate-resistant PCa (CRPC) to various cancer therapeutics. Elevated levels of P-Smad1/5/8, induced by suppression of TGF-β signaling, may also play a pivotal role in reversing the growth suppressive effects of Akt/mTOR antagonists. Exploration of this possibility and defining the underlying mechanisms involved are likely to have pivotal therapeutic implications.
